# MYC and RAS are unable to cooperate in overcoming cellular senescence and apoptosis in normal human fibroblasts

**DOI:** 10.1080/15384101.2018.1553339

**Published:** 2018-12-17

**Authors:** Fan Zhang, Siti Mariam Zakaria, Vedrana Högqvist Tabor, Madhurendra Singh, Susanna Tronnersjö, Jacob Goodwin, Galina Selivanova, Jiri Bartek, Alina Castell, Lars-Gunnar Larsson

**Affiliations:** a Department of Microbiology, Tumor and Cell Biology, Karolinska Institutet, Stockholm, Sweden; b Department of Medical Biochemistry and Biophysics, Karolinska Institutet, Stockholm, Sweden; c Danish Cancer Society Research Center, Copenhagen, Denmark

**Keywords:** MYC, RAS, p53, cellular senescence, apoptosis, BJ cells

## Abstract

The MYC and RAS oncogenes are sufficient for transformation of normal rodent cells. This cooperativity is at least in part based on suppression of RAS-induced cellular senescence by MYC and block of MYC-induced apoptosis by RAS – thereby canceling out two main barriers against tumor development. However, it remains unclear whether MYC and RAS cooperate in this way in human cells, where MYC and RAS are not sufficient for transformation. To address this question, we established a combined Tet-inducible H-RAS^V12^ and hydroxytamoxifen-inducible MycER system in normal human BJ fibroblasts. We show here that activation of RAS alone induced senescence while activation of MYC alone or together with RAS triggered DNA damage, induction of p53 and massive apoptosis, suggesting that RAS cannot rescue MYC-induced apoptosis in this system. Although coexpression with MYC reduced certain RAS-induced senescence markers (histone H3 lysine 9 trimethylation and senescence-associated β-GAL activity), the induction of the senescence marker p16INK4A was further enhanced and the culture ceased to proliferate within a few days, revealing that MYC could not fully suppress RAS-induced senescence. Furthermore, depletion of p53, which enhanced proliferation and rescued the cells from RAS-induced senescence, did not abrogate MYC-induced apoptosis. We conclude that MYC and RAS are unable to cooperate in overcoming senescence and apoptosis in normal human fibroblasts even after depletion of p53, indicating that additional oncogenic events are required to abrogate these fail-safe mechanisms and pave the way for cellular transformation. These findings have implications for our understanding of the transformation process in human cells.

**Abbreviations and acronyms**: CDK: Cyclin-dependent kinase; DDR: DNA damage response; DOX: Doxycycline; EdU: 5-ethynyl-2ʹ-deoxyuridine; FACS: Fluorescence Activated Cell Sorting; MycER: MYC-estrogen receptor; OHT: 4-hydroxytamoxifen; OIS: Oncogene-induced senescence; PP2A: Protein phosphatase 2A; ROS: Reactive oxygen species; SA-β-GAL: Senescence-associated β-galactosidase; SAHF: Senescence-associated heterochromatin foci; shRNA: Short hairpin RNA; YFP: Yellow fluorescent protein

## Introduction

*MYC* and *RAS* are two of the most important oncogenes, both highly implicated in tumorigenesis. The *MYC* oncogene family (*MYC, MYCN* and *MYCL*) encode transcription factors that control the expression of a large number of genes involved in processes relevant for tumorigenesis, including cell growth, apoptosis, metabolism, immortalization, differentiation and stem cell function [1–3]. Deregulation of *MYC* expression can be caused by chromosomal translocations or amplifications involving the *MYC* loci, or alternatively by perturbations in upstream regulators of MYC transcription or degradation. The *RAS* gene family (*HRAS, KRAS* and *NRAS*) encode membrane-bound GTPases that transduce extracellular signals from growth factor receptors to activate downstream effectors in various compartments of the cell leading to enhanced cell proliferation [4]. Activating point mutations or amplifications of *RAS*-family genes are very frequent in human cancers, such as pancreatic, colorectal and lung cancer [4].

Like other oncogenes, activated *MYC* and *RAS* trigger intrinsic tumor suppressor mechanisms that limit their tumorigenic potentials. Oncogenic *RAS* primarily triggers premature cellular senescence [5] – a state characterized by permanent cell growth arrest under which cells remain metabolically active [6–8]. Senescence is known to occur in normal cells during the aging process as a result of telomere erosion, but it can also be induced prematurely by a variety of different types of acute stresses, e.g. UV irradiation and other DNA-damaging agents, hypoxia, toxins or overactive oncogenes like RAS. The latter is called oncogene-induced senescence (OIS) and is caused for instance by replicative stress and generation of reactive oxygen species (ROS) as a result of overstimulation of proliferation and cellular metabolism. This causes DNA damage that triggers the DNA damage response (DDR) leading to increased levels and activation of the tumor suppressor p53 [6,7,9]. p53 activates genetic programs involved in apoptosis, DNA repair, cell cycle arrest and senescence. The latter involves induced expression of the cyclin-dependent kinase (CDK) inhibitor p21CIP1 (p21) [10], which blocks the activity of cyclin E/A/CDK2. OIS is also associated with induction of the CDK-inhibitor p16INK4a (p16) [5–8], which inhibits cyclin D/CDK4/6. Cyclin E/CDK2 and cyclin D/CDK4/6 complexes cooperate in phosphorylation and deactivation of the tumor suppressor protein pRB, which suppresses transcription of cell cycle genes regulated by the transcription factor E2F [11]. Induction of p21 and p16 will therefore together block CDKs targeting pRb, and this is considered a major mechanism by which p53 and pRB cooperatively shut down the cell cycle and induce senescence [6–8].

Activated *MYC*, on the other hand, primarily induces apoptosis [2,3,12]. This operates, at least in part, via MYC-induced expression of p19ARF (ARF), which inhibits the E3 ubiquitin ligase MDM2 that targets p53, thereby leading to p53 stabilization and induction of pro-apoptotic genes such as *BAX, PUMA* and *NOXA* [10]. MYC is also directly involved in activation of the mitochondrial apoptosis pathway by suppression of the anti-apoptotic genes *BCL-X_L_* and *BCL-2*, and the activation of the pro-apoptotic gene *BIM* in a p53-independent manner, and also sensitizes cell to apoptotic signals through the death receptor pathway [2,3].

It is well-known from the literature that MYC and RAS cooperate in tumorigenesis. Co-expression of oncogenic MYC and RAS enforces cell cycle progression and is sufficient to transform primary rodent cells [3,13,14]. Further, activated MYC and RAS or the downstream RAS effector BRAF synergistically induce tumor development *in vivo* in various transgenic mouse tumor models [15–21]. The basis for this cooperativity between MYC and RAS is still not well understood. RAS has been found to suppress MYC-induced apoptosis in rodent cells [22,23]. We and others had also shown previously that MYC is able to suppress activated RAS- and BRAF^V600E^-induced senescence in primary rodent cells in culture as well as BRAF^V600E^-induced senescence in transgenic mouse tumor models, which was linked to upregulation of cell cycle genes and repression of p16 and p21 [13,15,20]. Further, these studies showed that MYC inactivation restored RAS/BRAF-induced senescence, indicating that continuous MYC signaling is necessary for RAS/BRAF-induced tumorigenesis and tumor progression. Combined activation of the RAS and MYC pathways therefore seems to block two main anti-tumorigenic mechanisms in the cell – apoptosis and cellular senescence – that may, at least in part, explain the basis for the MYC/RAS cooperativity observed in the rodent system.

However, activation of MYC together with RAS is not sufficient for transformation of primary human cells [24]. This requires additional oncogenic events, including activation of hTERT, inactivation of the p53 and pRb pathways and deregulation of the PI3 kinase/AKT/protein phosphatase 2A (PP2A) pathway, although the exact requirements seem to be cell type- or context-dependent [25–34]. While abrogation of oncogene-induced apoptosis and senescence are required but not sufficient for transformation of human cells, it remains unclear whether MYC and RAS cooperate to suppress these fail-safe mechanisms in human cells in a similar way as in rodent cells. To address this question, we utilized normal human diploid BJ fibroblasts transduced with tetracycline-regulated H-Ras^V12^ and/or 4-hydroxytamoxifen (OHT)-controlled MycER expression/activation system. Our results show that doxycycline (DOX)-induced expression of activated RAS triggers senescence while OHT-induced activation of MycER led to increased cell death by apoptosis in agreement with previous reports [35,36]. Surprisingly, co-expressing both activated oncogenes in a dual inducible system led to similar levels of MYC-induced apoptosis, while RAS-induced senescence was not fully rescued by MYC. Further, depletion of p53 by shRNA did not promote MYC and RAS cooperativity. This suggests that MYC and RAS are unable to cooperate in human normal fibroblasts with respect to suppression of apoptosis and senescence and that additional oncogenic events are required before such cooperativity can be established.

## Results

### Induction of oncogenic RAS in human BJ fibroblasts leads to increased proliferation followed by growth arrest and senescence

**Figure 1.**
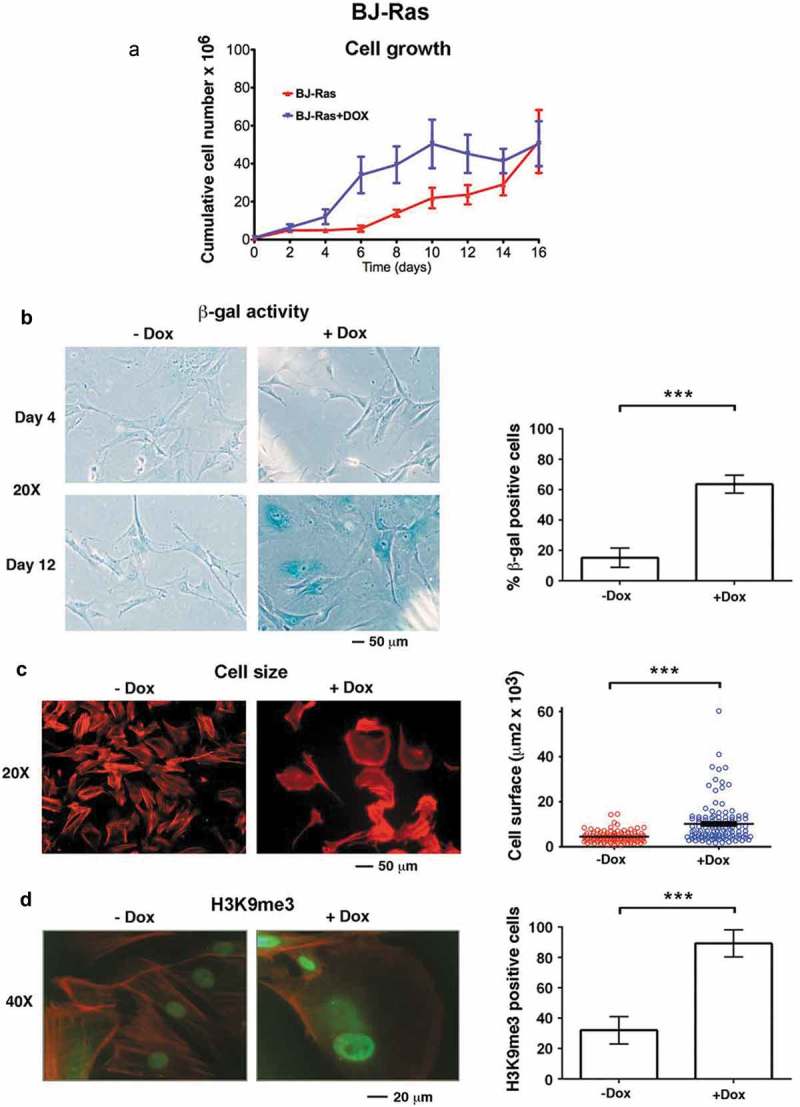
Doxycycline-induced H-RAS^V12^ expression induces cellular senescence in normal BJ human fibroblasts. BJ cells stably transduced with Tet-regulatable H-RAS^V12^ (BJ-Ras) cells were induced by doxycycline (DOX) assayed for senescence using different markers. (a) Cumulative cell growth of BJ-Ras cells induced by DOX or vehicle during a period of 16 days. Cell number at each time point was measured in triplicates. (b) Senescence associated β-GAL (SA-β-GAL) staining of BJ-Ras induced by DOX or vehicle for 4 and 12 days. Right panel; quantification of the percentage of β-GAL stained cells at 16 days after start of treatment. (c) Measurement of cell size by phalloidin staining after treatment with DOX or vehicle for 16 days. Right panel; quantification of cell area. (d) Immunofluorescence staining of H3K9me3 after treatment with DOX or vehicle for 16 days. Right panel; quantification of percentage of H3K9me3-positive cells. (a–d) Cells were split and reseeded every fourth day after treatment if necessary, and were given fresh media containing vehicle or DOX. Statistical data are presented as mean ± SEM, ***p < 0.001.

To study RAS-induced senescence in human primary cells, we used normal human BJ fibroblasts stably transduced with activated H-RAS^V12^ under the control of Tet-inducible promoter (henceforth called BJ-Ras) [35,36]. Stimulation of the cells with DOX led to enhanced growth compared to vehicle-only treated cells between day 4 and 10 (Figure 1(a)). This was followed by growth arrest from day 12 and onwards consistent with senescence induction, while untreated cells continued to proliferate, in agreement with previous reports [35,36].

To monitor senescence induction following RAS activation, senescence-associated β-galactosidase (SA-β-GAL) activity, cell size and senescence-associated heterochromatin foci (SAHF) – three well-established markers of senescence – were measured. Few cells stained positive for (SA-β-GAL) activity during the proliferative phase at day 4 but SA-β-GAL positivity increased at day 12 in DOX-treated but not in untreated cultures, coinciding with the onset of growth arrest (Figure 1(a,b)). By day 16 around 65 % of DOX-treated cells were SA-β-GAL positive compared to 15% of the control cells (Figure 1(b)). We also found a 2.3-fold increase in the mean cell size of DOX-treated cells (10,570 µm^2^) compared to control cells (4520 µm^2^), as determined by F-actin staining (Figure 1(c)). Further, DOX stimulation increased the percentage of cells that showed intense H3K9me3 staining (90 % compared to 30 % in the control), appearing as SAHF or a more widespread nuclear staining (Figure 1(d)). DOX treatment did not induce senescence in parental BJ cells used as controls, as determined by SA-β-GAL staining and monitoring of cell proliferation (Suppl. Figure S1).

Taken together, these observations confirm that DOX-induced expression of H-RAS^V12^ triggers senescence in the normal human BJ fibroblasts.

### Induction of RAS regulates expression of proliferation and senescence markers in BJ-Ras cells

**Figure 2.**
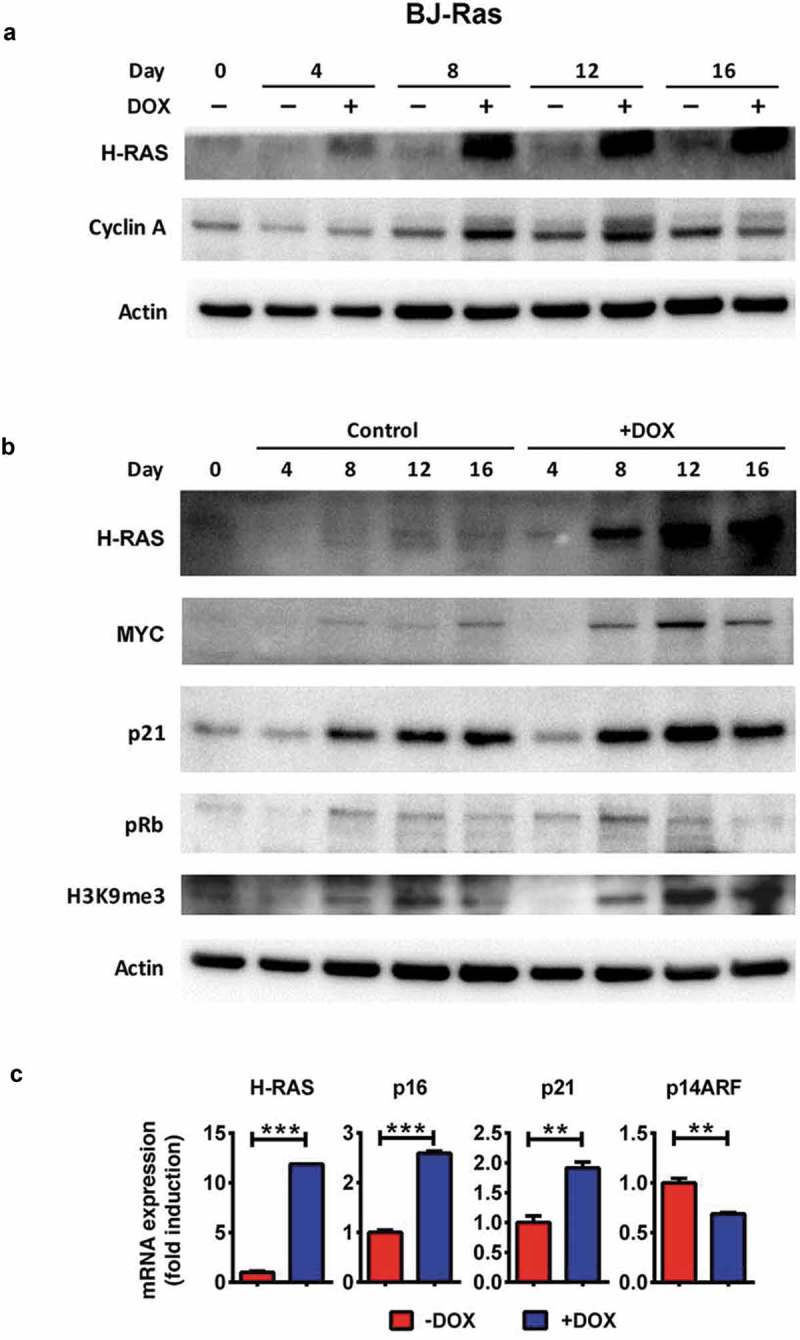
Expression of proteins/genes involved in cell cycle and senescence regulation upon H-RAS induction in BJ-Ras cells. (a) Immunoblot analysis of H-RAS and cyclin A1 after treatment of the cells with DOX or vehicle at indicated time points. (b) Immunoblot analysis of H-RAS, MYC, p21CIP1, phosphorylated pRb, H3K9me3 after treatment with DOX or vehicle at indicated time points. β-actin was used as loading control. (c) RT-qPCR analysis of mRNA expression of H-RAS, p16INK4A, p21CIP1 and p14ARF at day 16 after treatment with DOX or vehicle. Relative fold changes of mRNA expression are presented as mean ± SEM, **p < 0.01, ***p < 0.001. (a–c) The cells were split and reseeded during the experiments as described in Materials and Methods and in the legend to Figure 1.

We next investigated the expression of proliferation and senescence markers at the molecular level after DOX-treatment of BJ-Ras cells. Expression of the H-RAS protein increased continuously after DOX treatment reaching a maximum between 8–12 days, after which it remained stably expressed throughout the 16-day experiment (Figure 2(a,b), and Suppl. Figure S2). Further, a more than 10-fold increase in H-*RAS* mRNA expression occurred after DOX treatment (Figure 2(c)). The expression of the S/G2-phase marker cyclin A1 and of MYC increased gradually in DOX-treated cells with a maximum at day 12 (Figure 2(a,b) and Suppl. Figure S2), after which the levels of the proteins declined correlating with the growth retardation and senescence. In control cells, the expression of MYC and cyclin A, which also increased somewhat during the course of the experiment, and was maintained between day 8 and day 16 (Figure 2(a,b) and Suppl. Figure S2).

We also assessed several senescence markers at the molecular level. The level of p21 protein increased from day 8 onwards in both DOX-induced and uninduced cells, but reached higher levels in induced cells (Figure 2(b) and Suppl. Figure S2), corresponding to the increased p21 mRNA level (Figure 2(c)). Further, the phosphorylated (inactive) form of pRb peaked at 8 days after DOX treatment and then declined at day 16, indicating that pRb was reactivated again after the proliferative phase, as the cells approached senescence. In contrast, pRb phosphorylation was only slightly reduced in uninduced cells after day 8 (Figure 2(b) and Suppl. Figure S2). We also found markedly higher H3K9me3 levels in RAS-induced compared to uninduced cells, in particular from day 12 (Figure 2(b) and Suppl. Figure S2), as well as increased expression of p16 mRNA (Figure 2(c)). In agreement with a previous report [35], the level of ARF did not increase.

Taken together, these results suggest that induction of H-RAS^V12^ expression by DOX treatment in BJ-Ras cells triggers a proliferative phase that is followed by oncogene-induced senescence.

### Establishment of a stable dual-inducible BJ-Ras/MycER cell system

**Figure 3.**
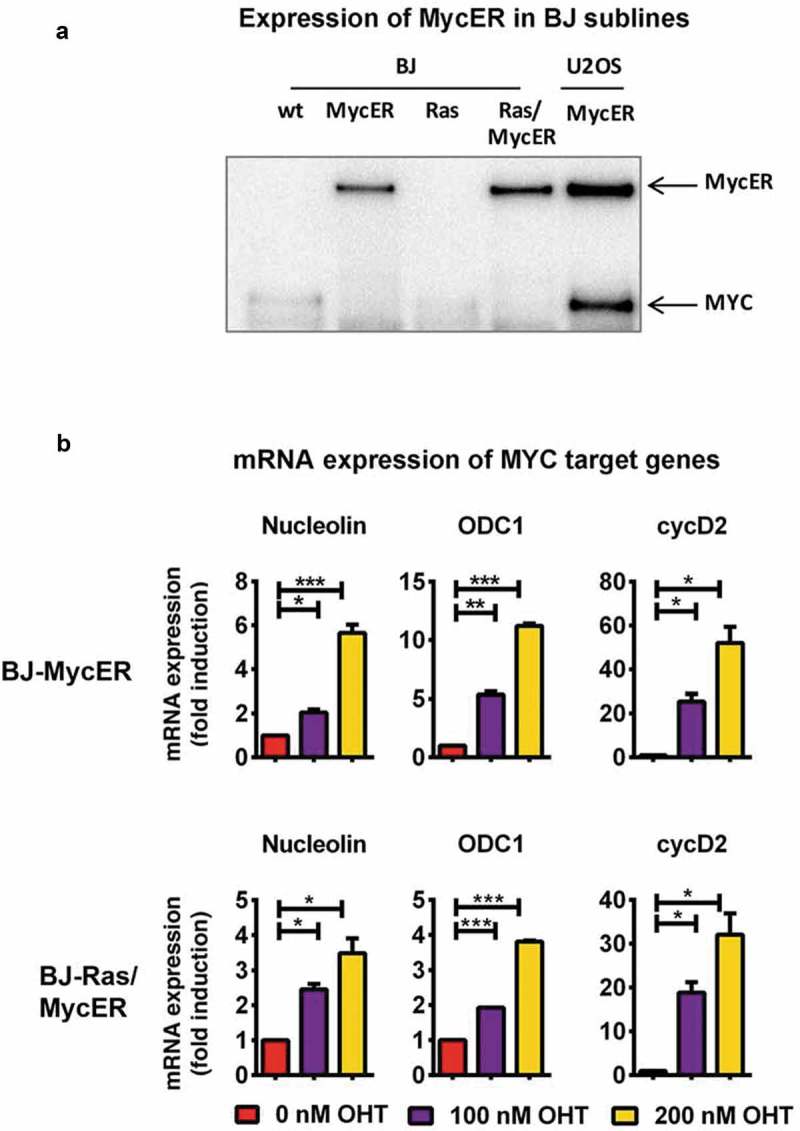
Establishment of BJ and BJ-Ras sublines expressing the 4-OH-tamoxifen regulatable MycER vector (BJ-MycER and BJ-Ras/MycER cells, respectively). (a) Immunoblot analysis of MycER protein expression (upper band) in selected BJ-MycER and BJ-Ras/MycER clones. U2OS osteosarcoma cells expressing the MycER construct was used as control. (b) RT-qPCR analysis of mRNA expression of MYC target genes after the cells were serum starved for 24 hrs and subsequently treated with either with 100 or 200 nM 4-OH-tamoxifen (OHT) for another 24 hrs. Relative fold change of mRNA expression is displayed as mean ± SEM, *p < 0.05, **p < 0.01, ***p < 0.001.

To investigate MYC and RAS cooperation in normal human cells, we created a cellular model system for dual induction of MYC and RAS. For this purpose, a retroviral vector encoding a MYC-estrogen receptor (MycER) fusion protein and the yellow fluorescent protein (YFP) reporter protein was used. MycER is regulated by the ER ligand 4-hydroxytamoxifen (4-OHT) [37]. BJ-Ras and parental BJ cells were transduced with the vector and BJ-Ras/MycER and BJ-MycER clones were subsequently isolated by FACS sorting of YFP positive cells (Suppl. Figure S3(a)). DNA integration and protein expression of the MycER construct was verified by PCR and immunoblot analyses in both BJ-MycER and BJ-Ras/MycER cells (Figure 3(a) and Suppl. Figure S3(b)).

Activation of the MycER protein by the addition of 4-OHT resulted in a dose-dependent upregulation of the well-known direct MYC target genes nucleolin, ODC1 and cyclin D2 both in the BJ-MycER and BJ-Ras/MycER cells, thereby verifying the functionality of MycER system (Figure 3(b), and Suppl. Figure S3(c)).

### MYC and RAS do not cooperate to overcome apoptosis and senescence in BJ cells

**Figure 4.**
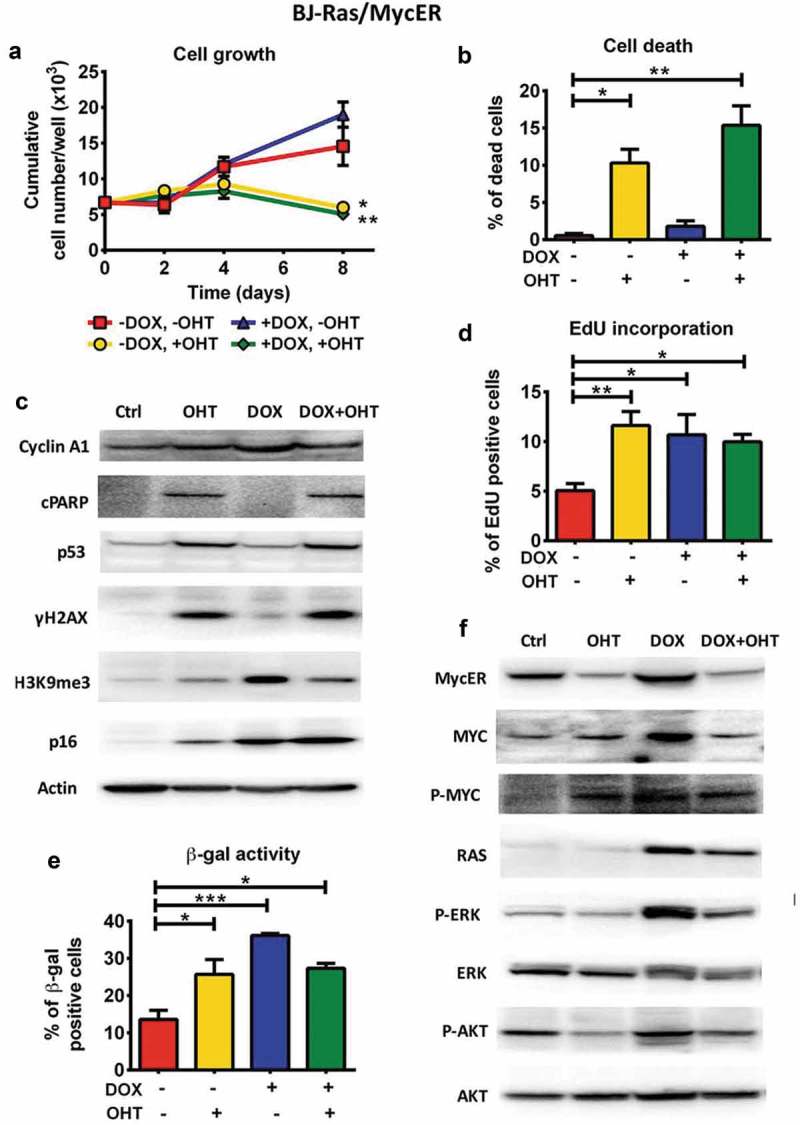
Combined activation of MYC and RAS in BJ-Ras/MycER cells does not rescue cells from RAS-induced senescence or MYC-induced apoptosis. BJ-Ras/MycER cells were treated with DOX (2 µg/ml), OHT (200 nM), DOX (2 µg/ml) + OHT (200 nM) or vehicle for 8 days (a, b, c, d and f) or 16 days (e). Assays were performed in triplicates. (a) Cumulative cell growth of BJ-Ras/MycER cells induced by indicated treatments as determined by trypan blue staining. (b) Measurement of cell death by trypan blue exclusion. (c) Immunoblot analysis of expression of cyclin A1, cleaved PARP (cPARP), p53, γH2AX, H3K9me3 and p16 as indicated. Note that the PARP1 antibody used (#9546) is specific for cleaved PARP. (d) Measurement of the percentage of S-phase cells by EdU incorporation assay using fluorescence microscopy. (e) SA-β-GAL assay performed at day 16 after indicated treatments. (f) Immunoblot analysis of expression of MYC, MycER, H-RAS, ERK, AKT and phosphorylated forms of these proteins as indicated. (c,f) Note that the immunoblots in (c) and (f) are from the same experiment. The actin loading control for both panels is provided in (c). (a–f) The cells were split and reseeded during the experiments as described in Materials and Methods and in the legend to Figure 1. Statistical data are presented as mean ± SEM, *p < 0.05, **p < 0.01, ***p < 0.001.

We next activated MYC and RAS in the BJ-Ras/MycER cells by treating the cells with OHT and DOX, respectively, either alone or simultaneously. After OHT stimulation alone, the growth rate of the culture started to decline after day 2 compared with control cells, and the net cell number decreased between day 4 and day 8 (Figure 4(a)). This was associated with strong induction of cell death (Figure 4(b)). In contrast, in cultures activated by RAS alone cell proliferation accelerated during the first 8 days, as expected. Importantly, simultaneous combined activation of RAS and MYC had a similar effect as MYC activation alone, and led to a net decrease in cell number between day 4 and day 8 (Figure 4(a)), again as a result of MYC-induced cell death. Quantification of cell viability showed 10% and 15% of dead cells in the MYC-activated and combined MYC+RAS-activated BJ-Ras/MycER cultures, respectively, by day 8 (Figure 4(b)), while there were only 2 and 1 % dead cells in RAS-induced and control cultures, respectively. OHT alone or in combination with DOX did not induce cell death in parental BJ or BJ-Ras cells used as controls, thus connecting the effects of OHT to the induced activation of MycER fusion protein (Suppl. Figure S4(a)). Further, neither of the treatments alone or in combination affected cell growth of parental BJ cells (Suppl. Figure S4(b)). We have shown previously that transduction of the empty vector did not affect OHT-responsiveness of parental BJ cells [36]. Monitoring apoptosis by measurement of cleaved PARP (cPARP) showed that both MYC induction alone and combined MYC+RAS resulted in a dramatic increase in cPARP in BJ-Ras/MycER cells, while RAS induction alone did not trigger PARP cleavage, suggesting that MYC activation induced apoptosis irrespective of RAS activity under these conditions (Figure 4(c) and Suppl. Figure S5(a)).

Measurement of DNA replication by EdU incorporation showed that activation of RAS alone, MYC alone and to a lesser extent the combined activation of MYC and RAS, led to an increase in the percentage of EdU positive cells (Figure 4(d)), indicating that MYC-activated cells replicated their DNA 8 days after induction, although this did not result in increased cell number due to induced cell death. The lower expression of cyclin A in the MYC-activated cells compared with the RAS-activated cells may also indicate that cell cycle progression of the former cells was interrupted (Figure 4(c) and Suppl. Figure S5(a)).

To investigate whether MYC-induced replication and apoptosis was linked to increased DNA damage, the expression the DDR marker γH2AX was measured. In contrast to RAS-activated cells, where only a slight increase in γH2AX was observed, a dramatic increase in γH2AX occurred after MYC activation alone or together with RAS. This was accompanied by induction of p53 (Figure 4(c) and Suppl. Figure S5(a)). These observations suggest that MYC activation triggers extensive DNA damage, resulting in increased level of p53 and subsequent induction of apoptosis. Apparently, concurrent RAS induction failed to block this process.

We next investigated senescence regulation in response to MYC activation alone or together with RAS. While RAS induction alone led to an increase in SA-β-GAL, H3K9me3 and p16 as expected, activation of MYC alone led to a more complex pattern; while there was a prominent increase in β-GAL positive cells, the expression of p16 and H3K9me3 was only modestly increased compared to their prominent induction by RAS (Figure 4(c,e) and Suppl. Figure S5(a)). Likewise, the dual induction of MYC and RAS led to the increase of β-GAL positive cells, although slightly less than after activation of RAS alone. OHT did not affect β-GAL activity in vehicle- or DOX-treated BJ-Ras cells (Suppl. Figure S5(c)), thus confirming that the effects of OHT in BJ-Ras/MycER cells are linked to the activation of MYC. Interestingly, RAS-induced expression of H3K9me3 was reduced by simultaneous MYC activation. In contrast, the expression of p16 was further enhanced upon combined activation of RAS and MYC (Figure 4(c) and Suppl. Figure S5(a)). MYC therefore seemed to be able to either enhance or dampen different aspects of RAS-induced senescence.

In summary, MYC activation induced DDR signaling, p53 activation, apoptosis and β-GAL activity regardless of RAS status. Further, MYC did not unambiguously suppress RAS-induced senescence. Conversely, RAS-activation did not suppress MYC-induced apoptosis, suggesting that MYC and RAS do not cooperate in the BJ cells in a manner expected based on their cooperation in rodent fibroblasts.

### MYC dampens the RAS pathway and RAS does not support MYC expression/activity in BJ cells coexpressing MYC and RAS

To find possible explanations for the lack of MYC and RAS cooperativity in BJ cells, we measured MYC and RAS expression and pathway activity in cells coexpressing MYC and RAS. As expected, DOX treatment alone increased expression of RAS and both endogenous MYC and the MycER protein (Figure 4(f) and Suppl. Figure S5(a)). The latter is in part the result of an increase in *MYC* mRNA (Suppl. Figure S5(b)) and in part probably due to stabilization of the MYC protein, consistent with the increased phosphorylation of MYC at Ser 62 (P-MYC), which is known to be involved in activation and stabilization of MYC [13,34,38–43]. As expected, DOX treatment also increased phosphorylation of the downstream effectors of RAS, ERK (P-ERK) and AKT (P-AKT) (Figure 4(f) and Suppl. Figure S5(a)). However, the combined DOX + OHT treatment led to somewhat reduced expression of RAS protein and mRNA, and marked downregulation of P-ERK and P-AKT compared with RAS induction alone (Figure 4(f) and Suppl. Figure S5(a,b), suggesting that MYC activation tuned down the RAS pathway. Furthermore, combined activation of MYC and RAS blunted the increased expression of MYC and MycER as well as the increased phosphorylation of MYC at Ser 62 occurring after RAS induction alone (Figure 4(f) and Suppl. Figure S5(a,b)), suggesting that RAS was not able to keep up MYC expression and phosphorylation when combined with MYC activation. Activation of MYC alone led to reduced mRNA expression of endogenous MYC and MycER. Since both OHT and DOX + OHT induced prominent activation of MYC targets genes (Figure 3(b) and Suppl. Figures S3(c), S5(b)), the reduction on MYC expression is likely due to negative autoregulation by activated MYC [44,45]. The expression of endogenous RAS and P-ERK was not affected by OHT treatment alone.

In summary, combined activation of MYC and RAS dampened the downstream effects of RAS signaling including phosphorylation of ERK and AKT, as well as the increased expression and phosphorylation of MYC mediated by RAS.

### RAS cannot rescue MYC-induced cell death even after tuning down or delaying MYC activation

Assuming that MYC-induced cell death is the main obstacle for MYC and RAS cooperativity in this system, we first tried to tune down induced MYC activity by reducing the concentration of OHT. However, even at 50 nM, a suboptimal dose that only partially induced MYC target gene expression (Suppl. Figure S3(c)), OHT could not restore cell proliferation when given alone or together with DOX (Suppl. Figure S5(d)). Considering that BJ cells undergo different growth phases after RAS induction – a proliferative phase during the first 8 days after stimulation followed by growth retardation and senescence starting from around day 8 – we explored whether activation of MYC at different time points after RAS induction would improve MYC/RAS cooperativity with respect to cell growth/viability. Suppl. Figure S5(e) shows that RAS-induction could not rescue cells from MYC-induced cell death at any of the time points. Even delaying MYC activation until 12 days post RAS induction, when cell growth was slowing down, nevertheless induced cell death and decreased cell number four days later compared with RAS-induction alone. Hence, RAS was not able to rescue cells from MYC-induced cell death irrespective of the magnitude or timing of MYC activation.

### p53 depletion does not improve MYC and RAS cooperativity

**Figure 5.**
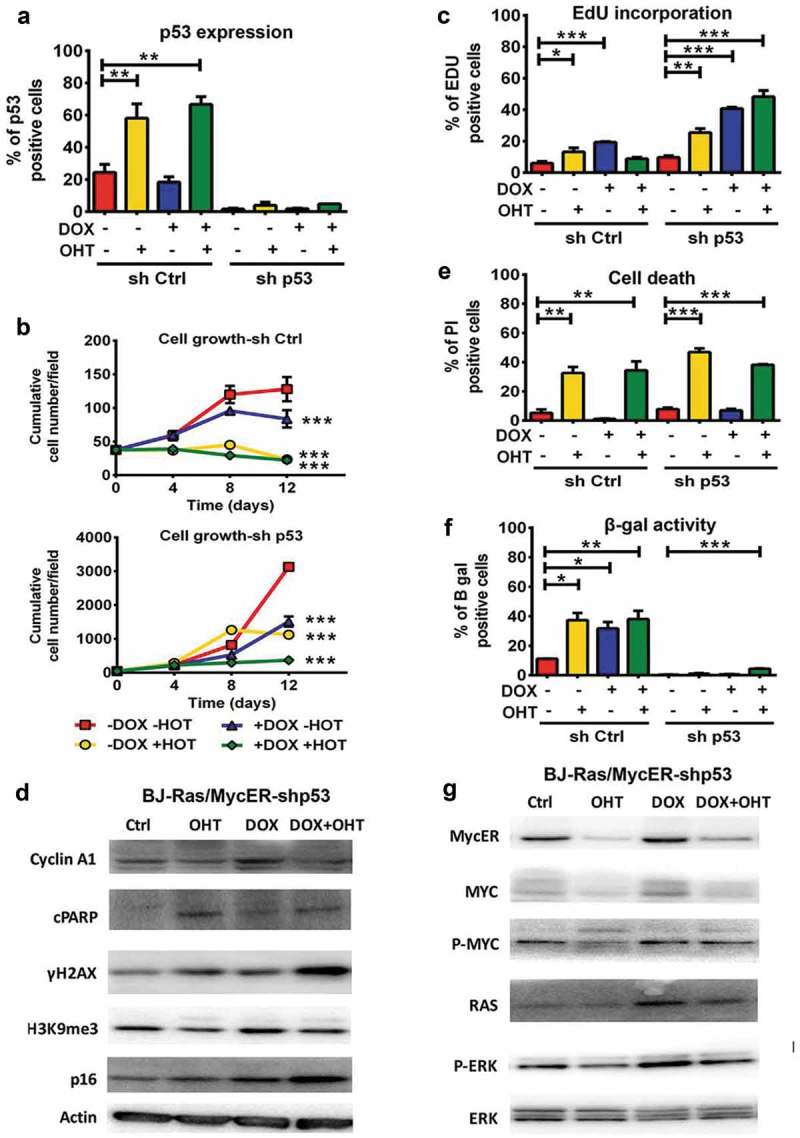
Depletion of p53 does not abrogate apoptosis after activation of MYC ± RAS in BJ-Ras/MycER-shp53 cells. BJ-Ras/MycER-shp53 and BJ-Ras/MycER-shCtrl cells were treated with DOX (2 µg/ml), OHT (100 nM), DOX (2 µg/ml) + OHT (100 nM) or vehicle for 16 days (b, g) or 8 days (c, e, f, h) as indicated. Assays were performed in triplicates. (a) Estimation of the percentage p53 positive cells in BJ-Ras/MycER-shp53 and BJ-Ras/MycER-shCtrl cultures after indicated treatments using fluorescence microscopy. (b) Growth of BJ-Ras/MycER-shCtrl and BJ-Ras/MycER-shp53 cells induced by indicated treatments during a period of 12 days by DAPI staining per field using fluorescence microscopy. (c) Measurement of the percentage of S-phase cells by EdU incorporation assay using fluorescence microscopy at day 8. (d) Immunoblot analysis of expression of proteins involved in the cell cycle, apoptosis, DNA damage and senescence (see legend to Figure 4) as indicated. (e) Measurement of cell death by propidium iodide staining at day 8. (f) SA-β-GAL assay was performed at day 16 after indicated treatments. (g) Immunoblot analysis of expression of MYC, RAS, ERK and phosphorylated forms of these proteins as indicated. d, g) Note that the immunoblots in (d) and (g) are from the same experiment. The actin loading control for both panels is provided in (d). (a–h) The cells were split and reseeded during the experiments as described in Materials and Methods and in the legend to Figure 1. Statistical data are presented as mean ± SEM, *p < 0.05, **p < 0.01, ***p < 0.001.

Considering the crucial role of p53 in oncogene-induced apoptosis and senescence, and the observed induction of p53 upon MYC activation (Figure 4(c) and Suppl. Figure S5(a)), we asked whether the cells could be rescued from MYC-induced cell death by p53 knockdown and thereby possibly improve MYC and RAS cooperativity. BJ-Ras/MycER sublines with p53 knockdown (BJ-Ras/MycER-shp53, referred to as “shp53”) and control (BJ-Ras/MycER-shCtrl, referred to as “shCtrl”) were established by lentiviral transduction of sh-p53RNA or scrambled shRNA vectors, respectively. Knockdown of p53 was verified by immunoblot (Suppl. Figure S6(a,b) and immunofluorescence, demonstrating that the percentage of p53-expressing cells after MYC activation was reduced from 60% in the shCtrl to around 5% in the shp53 cells (Figure 5(a)). We noted that untreated shp53 cells grew faster than the shCtrl cells (p < 0.001), which had a similar growth rate as parental BJ-Ras/MycER cells (Figures 5(b) and 4(a)). In contrast to the shCtrl cells, RAS-induced shp53 cells continued to grow after day 8 and onwards, suggesting that these had escaped RAS-induced senescence. Further, there was a marked enhancement of EdU incorporation in the shp53 cells compared to the shCtrl cells, in particular upon MYC and/or RAS activation (Figure 5(c)). Notably, the highest EdU incorporation was observed in the shp53 cells after dual induction of MYC and RAS, although the highest level of cyclin A1 was still observed after induction of RAS alone (Figure 5(d) and Suppl. Figure S7(a)). shp53 cells induced to activate MYC alone continued to proliferate beyond the point where shCtrl cells stopped growing (day 4), but ceased to grow after day 8 (Figure 5(b)). Importantly, despite the high EdU incorporation, there was no net increase in shp53 cell number from day 4 after combined MYC and RAS activation, likely due to cell death. Indeed, the percentage of dead cells remained high after MYC activation irrespective of RAS despite p53 knockdown, 40% and 50%, respectively, and was comparable to the percentage observed in shCtrl cells (Figure 5(e)). Induction of apoptosis was confirmed by the induction of cPARP in MYC-activated shp53 and shCtrl cells (Figure 5(d), Suppl. Figures S6 and S7). In addition, induction of γ-H2AX remained high after MYC activation and was even increased further after combined MYC and RAS induction in both in the shp53 and shCtrl cells (Figure 5(d), Suppl. Figures S6 and S7). This indicated that MYC-induced DNA damage and cell death could not be rescued by p53 knockdown.

With regard to senescence, the shp53 cells displayed a lower basal level of SA-β-GAL activity compared with the shCtrl and the parental BJ-Ras/MycER cells (Figure 5(f)). Notably, RAS-induced SA-β-GAL activity was almost completely abolished in p53 knockdown cells, which is in good agreement with their continuous proliferation (Figure 5(b)). Further, only a modest increase in the expression of p16 and H3K9me3 was observed in DOX-treated shp53 cells compared with shCtrl cells (Figure 5(d), Suppl. Figures S6 and S7), suggesting that p53 depletion inhibited senescence induced by RAS. Similarly, SA-β-GAL activity triggered by single activation of MYC was strongly suppressed in the shp53 cells and only a modest induction of p16 was observed and the level of H3K9me3 did not increase. The combined induction of MYC and RAS caused the largest increase in SA-β-GAL, but the number of SA-β-GAL positive shp53 cells was still low, only around 5% of the cells (Figure 5(f)). Further, while the expression H3K9me3 decreased slightly, there was a marked increase in p16 expression after combined activation of MYC and RAS in the shp53 cells compared to RAS induction alone (Figure 5(d), Suppl. Figure S7). This likely represents a subpopulation of senescent shp53 cells as also supported by the low percentage of SA-β-GAL positive cells (Figure 5(f)). Combined activation of MYC and RAS in the shCtrl cells led to strong reduction of H3K9me3 and increased p16 expression similar to the parental BJ-Ras/MycER cells (Figures 4(c), 5(d), Suppl. Figures S5(a), S6(c) and S7(b)). We conclude that the decreased cell number observed after co-induction of MYC and RAS in shp53 cells was likely due to increased cell death rather than senescence.

The DOX and/or OHT-regulated expression of MYC and RAS or the downstream ERK phosphorylation in observed in parental BJ-Ras/MycER cells and shCtrl cells was relatively similar in shp53 cells (Figures 4(f), 5(g), Suppl. Figures S5(a), S6(b) and S7).

In conclusion, p53 knockdown overrode RAS-induced senescence and promoted MYC- and RAS-induced S-phase entry, but largely failed to overcome MYC-induced apoptosis even in the presence of activated RAS, and thus did not improve MYC and RAS cooperativity in this system.

## Discussion

Early work showed that overexpression of MYC and activated RAS together are sufficient for oncogenic transformation of primary rodent cells [3,13,14]. However, the basis for this cooperativity has remained unclear. Previous work from our and other laboratories showed that MYC suppresses RAS-induced senescence while RAS inhibits MYC-induced apoptosis in rodent cells [13,22,23]. Since senescence and apoptosis are the two main barriers against tumor development, cooperativity between MYC and RAS in senescence and apoptosis suppression may thus constitute one of the major mechanisms behind the transformation of rodent cells by these oncogenes. However, MYC and RAS are not sufficient to transform primary human cells [24], since a number of additional control mechanisms are in place to block transformation of human cells [25–29,31–34]. While suppression of senescence and apoptosis are required but not sufficient for transformation of human cells [5,25,28,31–33], the question remains whether MYC and RAS cooperativity in overriding these two fail-safe mechanisms is valid and applies also to human primary cells.

To address this question, we introduced regulatable RAS and MYC systems into normal human BJ fibroblasts. Our results show that induction of expression of oncogenic RAS^V12^ alone by DOX treatment triggered senescence subsequent to an initial proliferative phase, while activation MycER alone by OHT induced apoptosis, as expected (Figures 1, 2 and 4). This is in agreement with previous observations in human primary cells upon overexpression of activated RAS or MYC, respectively [5,35,36,46]. Importantly, our results demonstrate that dual induction of MYC and RAS did not result in sustained proliferation but rather gave rise to a mixed population of apoptotic and senescent cells. Although there were very few apoptotic cells upon induction of RAS alone, RAS was apparently not able to rescue BJ cells from MYC-induced apoptosis (Figure 4(a–c), Suppl. Figure S5(a)). Further, the effect of MYC on RAS-induced senescence was quite complex; MYC clearly inhibited RAS-induced expression of the senescence marker H3K9me3 and slightly reduced SA-β–GAL-staining, suggesting that MYC was able to suppress some aspects of the senescent state (Figure 4(c,e) and Suppl. Figure S5(a)). However, the expression of the senescence marker p16 was further enhanced by the combined activation of MYC and RAS, and MYC could not rescue RAS-induced growth arrest but rather induced apoptosis. The regulation of the senescence program by MYC in BJ cells therefore seems to be more complex than in rodent cells. In conclusion, MYC and RAS do not cooperate in BJ cells with respect to suppression of senescence and apoptosis.

Previous studies of the requirements for senescence escape have shown that the viral oncogenes SV40 virus large T antigen (LT) and the adenovirus E1A are able to override RAS-induced senescence both in rodent and human cells. LT and E1A are known to block the Rb pathway and override p53-induced cell cycle arrest, thereby neutralizing the two main players in senescence regulation [5,25,27,28,31–33,47]. While MYC can substitute for LT and E1A in suppression of RAS-induced senescence in rodent fibroblasts, it apparently is unable to do so in human fibroblasts for unclear reasons, despite the fact that E1A induces apoptosis in a similar way as MYC [48]. One reason for this could be that MYC was unable to suppress RAS-induced p16 expression, but rather further enhanced it (Figure 4(c) and Suppl. Figure S5(a)). p16 is important for maintaining RB activity and thereby the senescent state. Down-regulation or loss of p16 has been shown to overcome RAS-induced senescence both in rodent and human cells [5,26,49,50]. Interestingly, Drayton et al reported that Leiden human fibroblasts deficient in p16 can be transformed by MYC and RAS together with hTERT without a need for further manipulations [26]. Apparently, MYC does not induce apoptosis in the Leiden cells, despite the induction of functional ARF and p53. Whether this phenomenon was connected to lack of p16 or some other alteration in these cells was not clarified in the article. Further, ectopic expression of RAS has been reported not to induce senescence in freshly isolated human neonatal foreskin fibroblasts, which express very low levels of p16 compared with BJ or other human primary fibroblasts known to senescence in response to RAS [51]. Prolonged cell culture of such cells immortalized by hTERT led to increased p16 expression, presumably due to cell culture stress, and acquired sensitivity to RAS-induced senescence, which could be reversed by p16 knockdown [51]. Interestingly, MYC has been shown to override activated BRAF-induced senescence and partially inhibit NRAS-induced senescence in human normal melanocytes, and depletion of MYC induced senescence in melanomas with activated BRAF/NRAS [52]. However, senescence induction in melanocytes and melanomas by these manipulations was reported to be p16- and p53-independent, suggesting that senescence regulation works differently in different cell types [52].

It is well documented that apoptosis and senescence are triggered in response to unrepairable DNA damage (review by [9]). RAS and MYC both cause DNA damage when over-activated as a result of ROS production and/or replication stress depending on the cellular context [3,6,7,35,36,46,53,54]. Indeed, as our data shows, both MYC and RAS activation led to increased levels of the DDR marker γH2AX at day 8 although this response was substantially stronger after activation of MYC (Figure 4(c) and Suppl. Figure S5A). In contrast to RAS, MYC also triggered a robust induction of p53. These differences could in part be due to different kinetics of DDR induction between MYC and RAS. Maya-Mendoza et al [36] showed previously that activation of MycER and RAS alone both triggered production of ROS, replication stress and γH2AX foci in BJ cells but with different kinetics – MycER already within 3–6 days and for RAS several days later at the end of the proliferative phase. Interestingly, the early induction of DDR by MYC triggered apoptosis while the late induction of DDR by RAS resulted in senescence. Importantly, our results showed that dual activation of MYC and RAS induced γH2AX and p53 to a similar level as MYC activation alone. RAS activation therefore was unable to rescue the cells from early MYC-induced DDR and apoptosis.

There could be several different explanations for the lack of cooperativity between MYC and RAS in the human BJ system. Conceivably, RAS and/or MYC signals might be either too weak or too strong or have an inadequate timing. Combined MYC and RAS activation resulted in a somewhat reduced RAS level and a marked decrease in ERK and AKT phosphorylation compared with RAS alone (Figure 4(f) and Suppl. Figure S5), suggesting that MYC somehow dampens RAS signaling in this system. In rodent cells, both the PI3K/AKT and MEK/ERK pathways have been implicated in inhibition of MYC-induced apoptosis by RAS [22,23]. The reduced ERK and AKT phosphorylation after combined MYC and RAS induction may therefore have resulted in insufficient PI3K/AKT and MEK/ERK anti-apoptotic signaling. MYC activation downstream of ERK in human triple negative breast cancer activates a negative feedback loop that downregulates several tyrosine kinase receptors, which impacts on RAS/MAPK/AKT signaling [55]. Possibly the MAPK/MYC negative auto-loop is stronger in human cells than in rodent cells.

Also the RAS-induced expression of MYC mRNA and protein as well as the increased phosphorylation of MYC at Ser 62 was blunted after combined activation of MYC and RAS (Figure 4(f) and Suppl. Figure S5). Since the effect of RAS on MYC gene expression is known to be mediated via activation of ERK [43,56], this reduction could therefore in part be due to the observed reduced ERK activity. Further, phosphorylation of MYC Ser-62 by ERK or CDKs has been shown to increase the activity and stability of the MYC protein [13,34,38–43], suggesting that part of the reduced MYC expression may occur at the protein level. Ser-62 phosphorylation seems to be particularly important in survival and regeneration in response to DNA damage and for suppression of senescence [13,39,41,42]. In addition, RAS may influence MYC protein stability via the PI3K/AKT pathway, which inhibits GSK3β/FBXW7-mediated proteasomal degradation of MYC through regulation of MYC Thr58 phosphorylation [40,41]. The reduced AKT activity observed after combined MYC and RAS activation may therefore result in increased turnover of MYC.

Since the expression of MYC target genes was induced as expected by both OHT alone and OHT+DOX, another explanation for the reduced MYC expression is that this involves a negative feed-back loop where active MYC downregulates its own expression [44,45]. Part of this negative feed-back regulation is known to occur at the protein level through regulation of MYC phosphorylation at the Ser-62 and Thr-58 residues in the N-terminus of MYC. A third explanation is that MYC expression is down-regulated as a result of DDR and apoptotic signaling triggered by MYC itself [40,57].

We also investigated whether tuning down MYC activity by lowering the OHT concentration or whether RAS pretreatment, which potentially could trigger anti-apoptotic signaling prior to MYC activation, would change the outcome. However, these measures did not improve the situation (Suppl. Figures S5(d) and S5(e)), suggesting that other oncogenic events are required in order to enable MYC and RAS cooperativity.

Considering the pivotal role of p53 in both DNA-damage response and apoptosis, and the strong induction of DDR, p53 and apoptosis upon MYC activation alone or together with RAS, we asked whether depletion of p53 could facilitate the collaboration of MYC and RAS in the BJ system. Although the growth rate of cells activated by MYC alone or together with RAS was improved in p53-depleted cells initially and a strong increase EdU-incorporating cells was observed, there was no net increase in the number of MYC+RAS activated cells already after day 4 (Figure 5(b,c)). This was mainly due to cell death, which was maintained at a similar level despite loss of p53 (Figure 5(d,e) and Suppl. Figure S7A). We therefore concluded that p53 knockdown did not improve MYC and RAS cooperativity in this system. As mentioned in the introduction, MYC can induce apoptosis also in a p53-independent manner, for instance by suppression of expression of the anti-apoptotic proteins BCL-X_L_ and BCL-2, activation of the pro-apoptotic protein BIM, and enhancing apoptotic signaling through the death receptor pathways [2,3,58].

Further, p53 depletion rescued cells from RAS-induced senescence irrespective of MYC status (Figure 5(b,d,f) and Suppl. Figure S7(a)). The requirement for loss of p53 or of pRb function, or of both, for senescence escape in normal human cells seems to be cell type and context-dependent [5,25,28,31,33]. Our results showing that loss of p53 is sufficient for senescence escape in BJ cells are consistent with previous reports from RAS-activated BJ cells where either RNAi or dominant negative p53 was used [25,33,35,47].

Taken together, our study shows that MYC and RAS, in contrast to rodent cells, were not able to collaborate to bypass senescence and apoptosis in human BJ cells, even after depletion of p53. MYC activation in this system caused severe cellular stress leading to DDR, p53 activation and apoptosis, which overruled MYC activities that in other contexts would support long term RAS-induced proliferation and senescence evasion. This suggests that additional oncogenic events are required not only for full transformation but also for senescence and apoptosis evasion in normal human cells.

## Materials and methods

### Cell culture

BJ human fibroblasts (ATCC), U2-OS MycER cells (kindly provided by Martin Eilers, University of Würzburg) and the Phoenix–Ampho retroviral packaging cell line (kindly provided by Garry Nolan, Stanford University, CA, USA) were cultured in DMEM supplemented with 10% FBS, 2 mM L-glutamine, 50 U/ml of penicillin and 50 mg/ml of streptomycin, and for Phoenix–Ampho cells in addition 1 mM sodium pyruvate. BJ cells with doxycycline (DOX)-inducible expression of Ras^V12^ (Lenti-X^TM^ Tet-On Advanced Inducible Expression System, Clontech) (BJ-Ras) were created by double lentivirus infection as previously described [35], and were maintained in culture media as above containing 0.5 μg/ml of puromycin (Sigma, P8833) and 100 μg/ml of G418 (Clontech, 631307). RAS^V12^ expression was induced using 2 μg/ml of doxycycline (Sigma, D9891). BJ-MycER and BJ-Ras/MycER cells were cultured in DMEM medium without phenol red (Gibco) supplemented with 10% FBS and antibiotics. MycER [37] activation was induced by 50–200 nM of 4-hydroxytamoxifen (4-OHT) (Sigma) dissolved in ethanol (Kemetyl).

### Expression vectors, production of viral particles and transduction of target cells

The following expression vectors were used in transfections: pMSCV-IRES-EYFP (Control), pMSCV-MycER-IRES-EYFP (MycER) [59], kindly provided by Alf Grandien, Karolinska Institute. Recombinant viral particles were produced as previously described [60] with slight modifications. Briefly, 10 µg of retroviral vectors were transfected into Phoenix-Ampho cells and incubated at 32°C. The supernatant was harvested 24 and 48 hours post-transfection, filtered through a 0.45 µM filter, and used directly or kept at −80°C in aliquots until required for viral transduction. For retroviral transduction, target cells were seeded at about 70% confluency and retroviral supernatants were applied together with 8 μg/ml of polybrene for 2 rounds of 24 hours infections at 32°C. Cells transduced with the MycER construct were expanded for 1 passage and then selected based on YFP positivity by Fluorescence Activated Cell Sorting (FACS) using a FACSVantage/DiVa equipped with a PowerMac G4 and a PC HP X4000 workstation.

### Lentivirus shRNA knockdown

The construct encoding the short hairpin RNA (shRNA) directed against p53 was kindly provided by Katerina Gurova (Buffalo, NY, USA). Lentiviruses were generated by transient transfection of HEK293T cells plated at a density of 5 × 10^6^ cells per T75 flask. The following day, cells were co-transfected with 3^rd^ generation helper vectors pCMV-VSVG, pMDLg-RRE, pRSV-REV and shRNA construct or control vector using the calcium phosphate precipitation method. Transfected cells were incubated for 4–6 h and followed by the incubation with fresh media for additional 48 h. Next, the medium was harvested and filtered through a 0.45 µm pore filter. For transduction, cells were plated at 50–60% confluence and incubated for 48 h with lentiviral supernatants containing 8 µg/ml Polybrene (Sigma-Aldrich).

### SA-β-GAL, cell proliferation and viability assays

SA-β-GAL staining of BJ-Ras cells was conducted using the Biovision senescence kit according to manufacturer’s protocol (#K320-250). Alternatively, multi-staining was performed as described previously [61], with slight modifications. Before harvesting, cells were incubated with 10 μM 5-ethynyl-2ʹ-deoxyuridine (EdU) (Thermo scientific) for 16 hours. Then, the cells were fixed by 2% formaldehyde (Histolab) and 0.2% glutaraldehyde (Sigma) for 5 min, followed by β-GAL working solution (1x citric acid/sodium phosphate buffer, 5 mM potassium ferricyanide, 150 mM NaCl, 2 mM MgCl_2_ and 1 mg/ml X-GAL at pH 6.0) (Sigma and Thermo scientific) for 8 hours. After permeabilization by 0.5% Triton X-100 (Sigma) for 5 min, samples were incubated with freshly prepared EdU incubation solution (1 mM CuSO_4_, 100 mM Tris, 2 μg/ml Fluorescent azide far red 647 and 100 mM ascorbic acid) (Sigma and Thermo scientific) for 30 min, and mounted with ProLong® Gold Antifade Mountant with DAPI (P36931) (Thermo scientific). Images of SA-β-GAL and multi-stained cells were taken using a Zeiss AxioVert 40C microscope equipped with a high-resolution AxioCam MRc5 camera and Axiovert software (Zeiss). To determine cell number and viability, cells were trypsinized and counted by trypan blue (Sigma) exclusion using BioRad cell counter, or alternatively, stained with 1 μg/ml propidium iodide (PI) (Sigma) and 1 μg/ml Hoeschst 33342 (Sigma) for 15 min and monitored by fluorescent microscopy.

For these experiments, exponentially growing cells were seeded in either 96-well plates (~2500 cells/well), 48-well plates (~6000 cells/well) or in 24-well plates (~12,000 cells/well) and then split and reseeded every 4^th^ day with fresh medium containing vehicle, DOX and/or OHT. The splitting factor is taken into account for the calculation of cumulative cell number.

### Immunofluorescence staining

Cells were fixed with 4% of PFA for 20 min and permeabilized by 0.5% Triton X-100 for 5 min. After blocking with 4% BSA for 1 hour, samples were incubated with primary antibody in blocking solution overnight. After washing, secondary antibody was incubated for 1 hour, and mounted with ProLong® Gold Antifade Mountant with DAPI (P36931) (Thermo scientific). The following primary antibodies were used: H3K9me3 (Millipore, 07–523) and p53 (DO-1, Santa Cruz Biotechnology). Alexa Fluor™ antibodies (Thermo Scientific) were used as secondary antibody. Phalloidin, used for staining of F-actin, was kindly provided by Pontus Aspenström, Karolinska Institutet. Images of stained cells were visualized using an inverted microscope (Zeiss Axiovert 200M) and captured using a Hamamatsu Orca-ER camera and Axiovert software (Zeiss). Automated fluorescent imaging and cell analysis were performed in ImageXpress® Micro (Molecular Devices).

### Immunoprecipitation and immunoblot analysis

The protein lysates were harvested in buffer containing 1% NP-40, 100 mM Tris pH 8, 150 mM NaCl, 5 mM EDTA (Sigma) and 50 KIE Trasylol (Bayer, 01511764). Complete tablet (Roche, 04693132001) and PhosStop (Roche, 04906837001) were added according to the manufacturer instructions. The lysates were incubated on ice for 30 min and spun at 13,000 rpm for 15 min. Supernatant was transferred to a new tube, snap frozen and stored at −80°C until samples at all time points were collected. Protein concentration was determined using Bradford assay (BioRad, 500–0006). Immunoprecipitation and immunoblot analyses were performed as described previously [38].

The following antibodies were used: MYC (sc-764), p21 (sc-6246), Cyclin A1 (sc-239), p27 (sc-528), p53 (sc-126), all from Santa Cruz Biotechnology, Actin (Sigma, A5441), RAS (61002) and p16 (554079), both from BD Bioscience, H3K9me3 (M07-523) and γH2AX (JBW301), both from Millipore, MYCN phospho-Ser54 (Bethyl Laboratories, A300-206A, which also recognizes MYC phospho-Ser62), phospho-AKT (#4691), AKT (#4060), phospho-ERK (#4370), ERK (#4695), cPARP (#9546) and Rb antibody sampler kit (#9969), all from Cell Signaling Technology. Quantification of the intensities of the bands in the western blots was performed by Image J (Rasband, W.S., ImageJ, U. S. National Institutes of Health, Bethesda, Maryland, USA, https://imagej.nih.gov/ij/, 1997–2018).

### RT-qPCR

RNA from cells were extracted by iScript™ RT-qPCR Sample Preparation Reagent (1708898, Bio-Rad) and cDNA was synthesized using RevertAid MYC Minus First Strand cDNA Synthesis Kit (K1631, ThermoFisher Scientific) according to manufacturer’s protocol. Quantitative real-time reverse transcription-PCR (RT-qPCR) was performed using iTaq Univer SYBR Green SMX 500 (1725121, Bio-Rad) on Applied Biosystem qPCR machine. For measurement of MycER-induced activation of MYC target genes, cells were serum starved for 24 hrs prior to OHT treatment in order to down-regulate expression of endogenous MYC and other factors that may contribute to the expression of these genes.

The following primers were used for RT-PCR:

H-RAS forward: GGCATCCCCTACATCGAGA

H-RAS reverse: CTCACGCACCAACGTGTAGA

MYC forward: GACTCCAGCGCCTTCTC

MYC reverse: CTTCCTCATCTTCTTGTTCCTCC

MycER forward: TTGCGGAAACGACGAGAACA

MycER reverse: AGGACAAGGCAGGGCTATTC

Nucleolin forward: AGGTGACCCCAAGAAAATGG

Nucleolin reverse: AGCCTTCTTGCCTTTCTTCTG

p16INK4A forward: GAAGGTCCCTCAGACATCCCC

p16 INK4A reverse: CCCTGTAGGACCTTCGGTGAC

GAPDH forward: ACATCGCTCAGACACCATG

GAPDH reverse: TGTAGTTGAGGTCAATGAAGGG

p21CIP1 forward: GGCAGACCAGCATGACAGATTTC

p21 CIP1 reverse: CGGATTAGGGCTTGG

p14ARF forward: CTACTGAGGAGCCAGCGTCT

p14ARF reverse: CTGCCCATCATCATGACCT

ODC1 forward: TCTGCTTGATATTGGCGGTG

ODC1 reverse: GGCTCAGCTATGATTCTCACTC

Cyclin D2 forward: GCTGGAGGTCTGTGAGGAAC

Cyclin D2 reverse: TCGGTGTAAATGCACAGCTT

### Genomic PCR

Genomic DNA was harvested from MYC-ER-transduced cells using the Flexi Gene DNA kit (Qiagen, 51204) and PCR was conducted using Taq polymerase (Fermentas) according to the respective manufacturer instructions. PCR products were run on 1% agarose gel in Tris-acetate-EDTA (TAE) buffer and detected in a Gel Doc XR+ System (BioRad).

The following PCR primer sequences were used for detection of genomic integration of the MycER construct:

MYC forward: CAGATCAGCAACAACCGAAA

ER reverse: AGGTGGACCTGATCATGGAG

### Statistical analysis

Proportional data corresponding to cell proliferation, viability and senescence assays and immunohistochemistry were analyzed with one-way ANOVA with post-hoc Dunnett’s multiple comparison test. Relative fold changes in mRNA expression were analyzed with student T-test or one-way ANOVA. All analyses were carried out in GraphPad Prism version 7.00 (for Windows, GraphPad Software, La Jolla California USA, www.graphpad.com), at a level of significance α = 0.05.
