# Supplementary material for: MYC and RAS are unable to cooperate in overcoming cellular senescence and apoptosis in normal human fibroblasts
Source: Cell Cycle. 2018 Dec 17;17(24):2697–715. doi: 10.1080/15384101.2018.1553339 (PMC6343716; doi:10.1080/15384101.2018.1553339)
Supplement: Supplemental Material [file kccy-17-24-1553339-s001.pptx]

## Slide 1
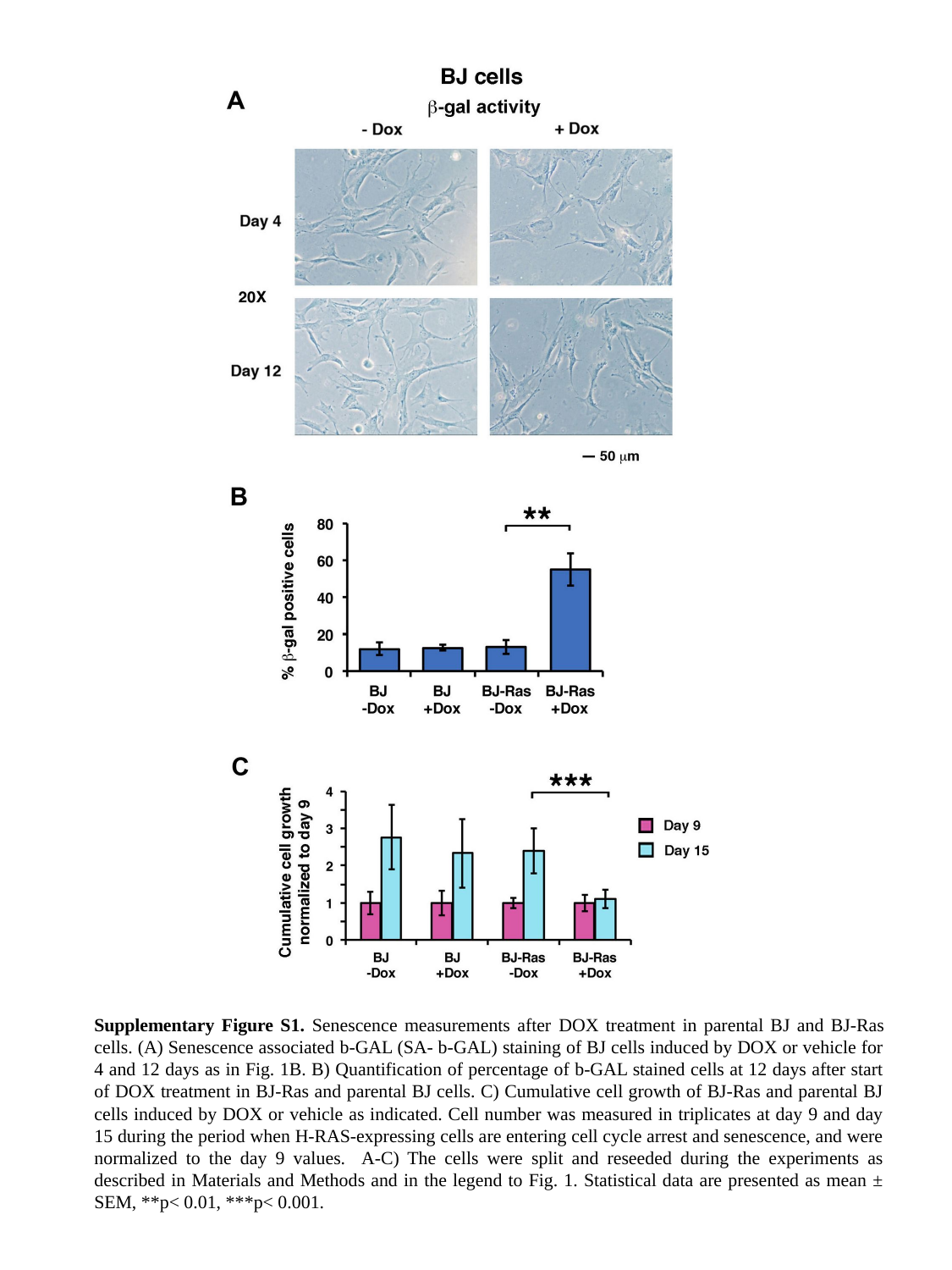

Supplementary Figure S1. Senescence measurements after DOX treatment in parental BJ and BJ-Ras cells. (A) Senescence associated b-GAL (SA- b-GAL) staining of BJ cells induced by DOX or vehicle for 4 and 12 days as in Fig. 1B. B) Quantification of percentage of b-GAL stained cells at 12 days after start of DOX treatment in BJ-Ras and parental BJ cells. C) Cumulative cell growth of BJ-Ras and parental BJ cells induced by DOX or vehicle as indicated. Cell number was measured in triplicates at day 9 and day 15 during the period when H-RAS-expressing cells are entering cell cycle arrest and senescence, and were normalized to the day 9 values. A-C) The cells were split and reseeded during the experiments as described in Materials and Methods and in the legend to Fig. 1. Statistical data are presented as mean ± SEM, **p< 0.01, ***p< 0.001.

## Slide 2
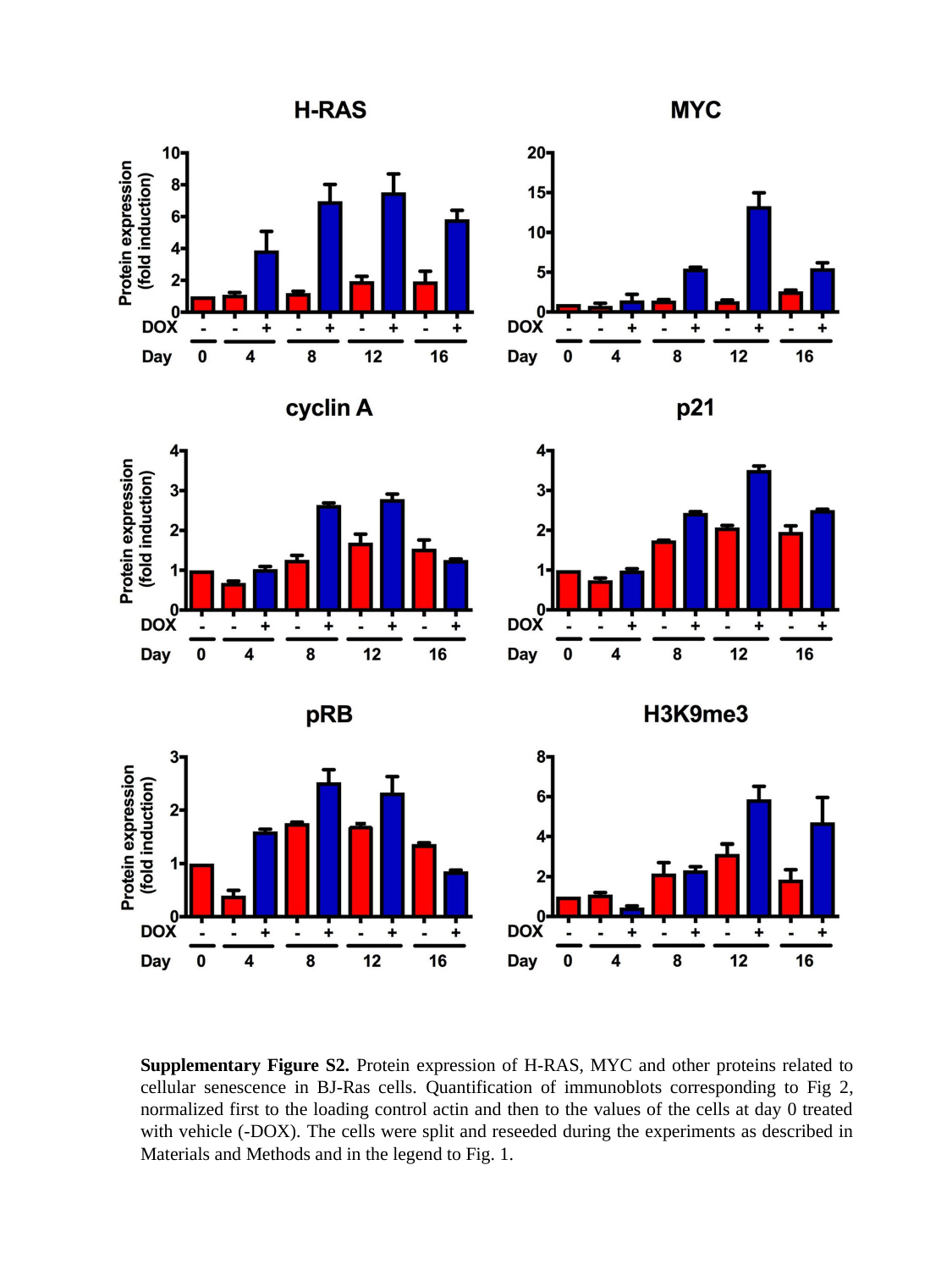

Supplementary Figure S2. Protein expression of H-RAS, MYC and other proteins related to cellular senescence in BJ-Ras cells. Quantification of immunoblots corresponding to Fig 2, normalized first to the loading control actin and then to the values of the cells at day 0 treated with vehicle (-DOX). The cells were split and reseeded during the experiments as described in Materials and Methods and in the legend to Fig. 1.

## Slide 3
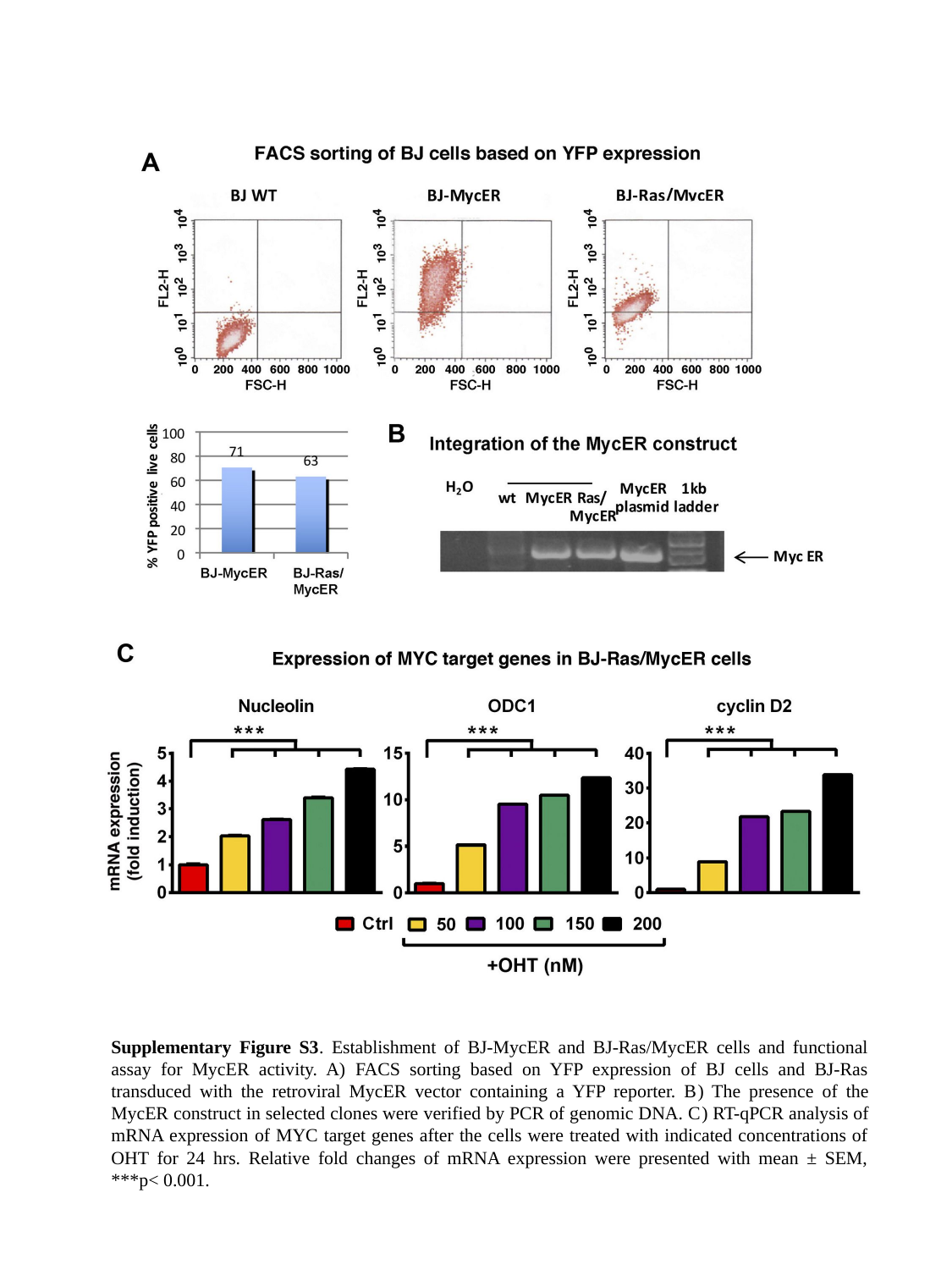

Supplementary Figure S3. Establishment of BJ-MycER and BJ-Ras/MycER cells and functional assay for MycER activity. A) FACS sorting based on YFP expression of BJ cells and BJ-Ras transduced with the retroviral MycER vector containing a YFP reporter. B) The presence of the MycER construct in selected clones were verified by PCR of genomic DNA. C) RT-qPCR analysis of mRNA expression of MYC target genes after the cells were treated with indicated concentrations of OHT for 24 hrs. Relative fold changes of mRNA expression were presented with mean ± SEM, ***p< 0.001.

## Slide 4
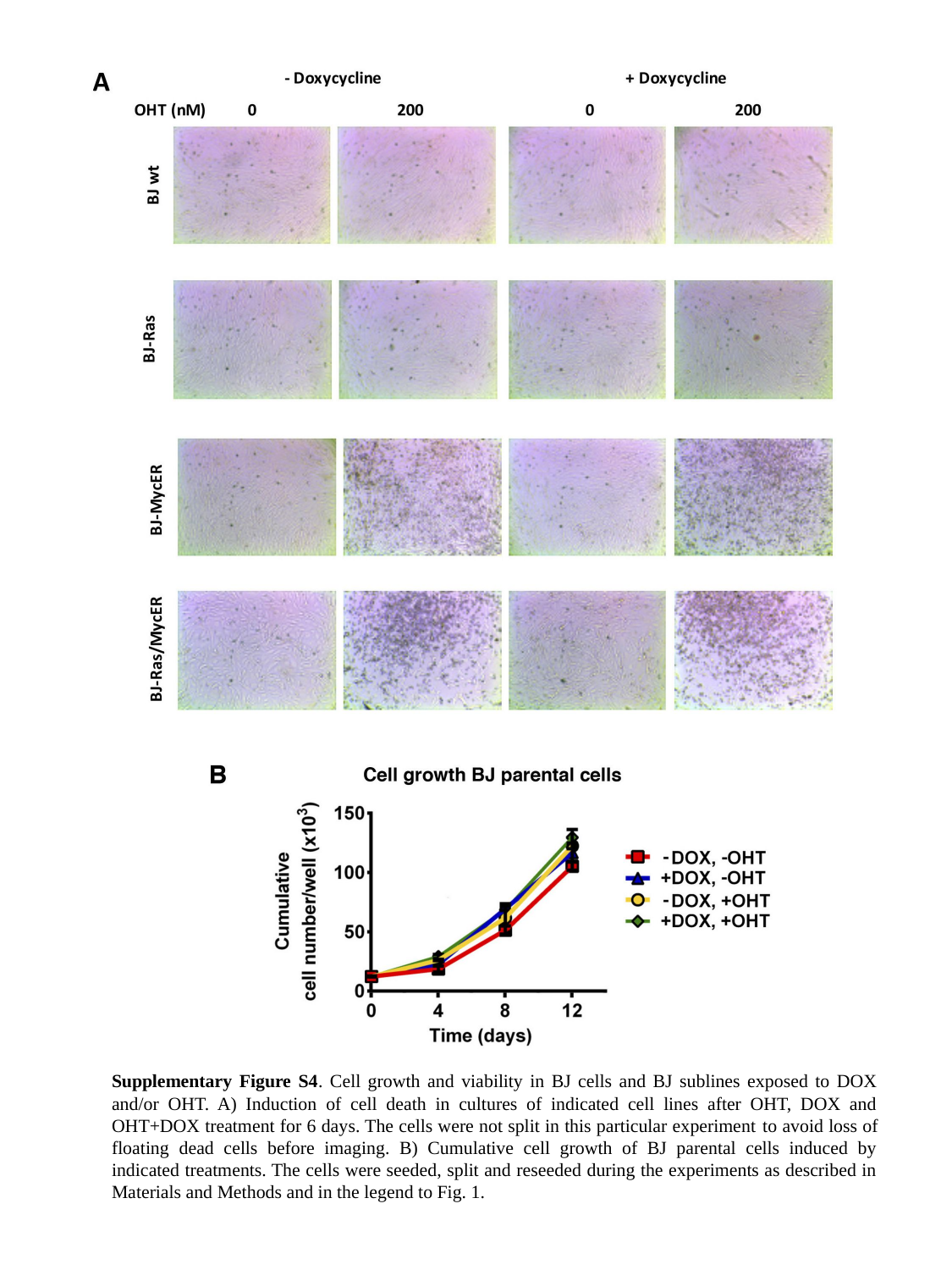

Supplementary Figure S4. Cell growth and viability in BJ cells and BJ sublines exposed to DOX and/or OHT. A) Induction of cell death in cultures of indicated cell lines after OHT, DOX and OHT+DOX treatment for 6 days. The cells were not split in this particular experiment to avoid loss of floating dead cells before imaging. B) Cumulative cell growth of BJ parental cells induced by indicated treatments. The cells were seeded, split and reseeded during the experiments as described in Materials and Methods and in the legend to Fig. 1.

## Slide 5
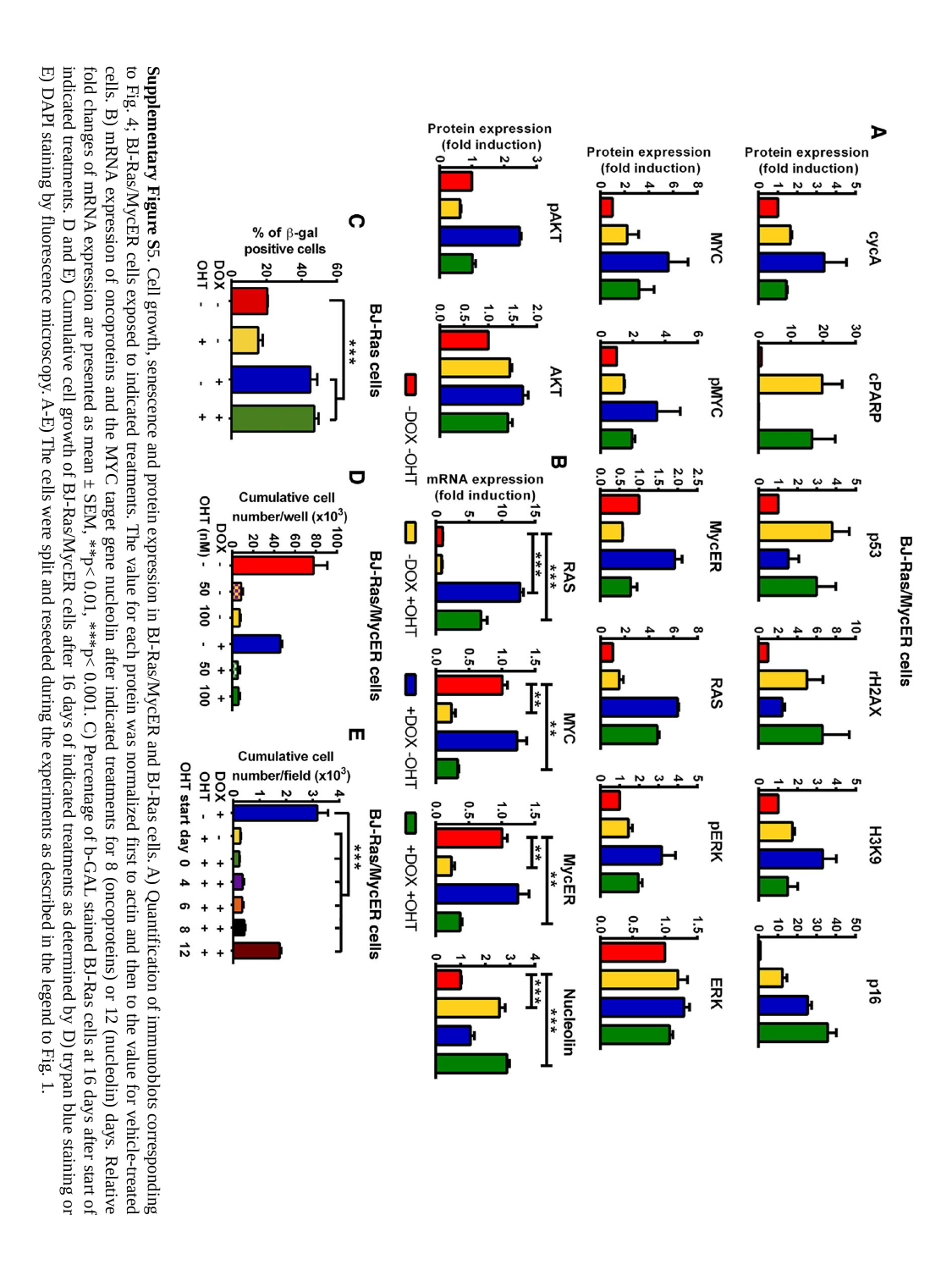

Supplementary Figure S5. Cell growth, senescence and protein expression in BJ-Ras/MycER and BJ-Ras cells. A) Quantification of immunoblots corresponding to Fig. 4; BJ-Ras/MycER cells exposed to indicated treatments. The value for each protein was normalized first to actin and then to the value for vehicle-treated cells. B) mRNA expression of oncoproteins and the MYC target gene nucleolin after indicated treatments for 8 (oncoproteins) or 12 (nucleolin) days. Relative fold changes of mRNA expression are presented as mean ± SEM, **p< 0.01, ***p< 0.001. C) Percentage of b-GAL stained BJ-Ras cells at 16 days after start of indicated treatments. D and E) Cumulative cell growth of BJ-Ras/MycER cells after 16 days of indicated treatments as determined by D) trypan blue staining or E) DAPI staining by fluorescence microscopy. A-E) The cells were split and reseeded during the experiments as described in the legend to Fig. 1.

## Slide 6
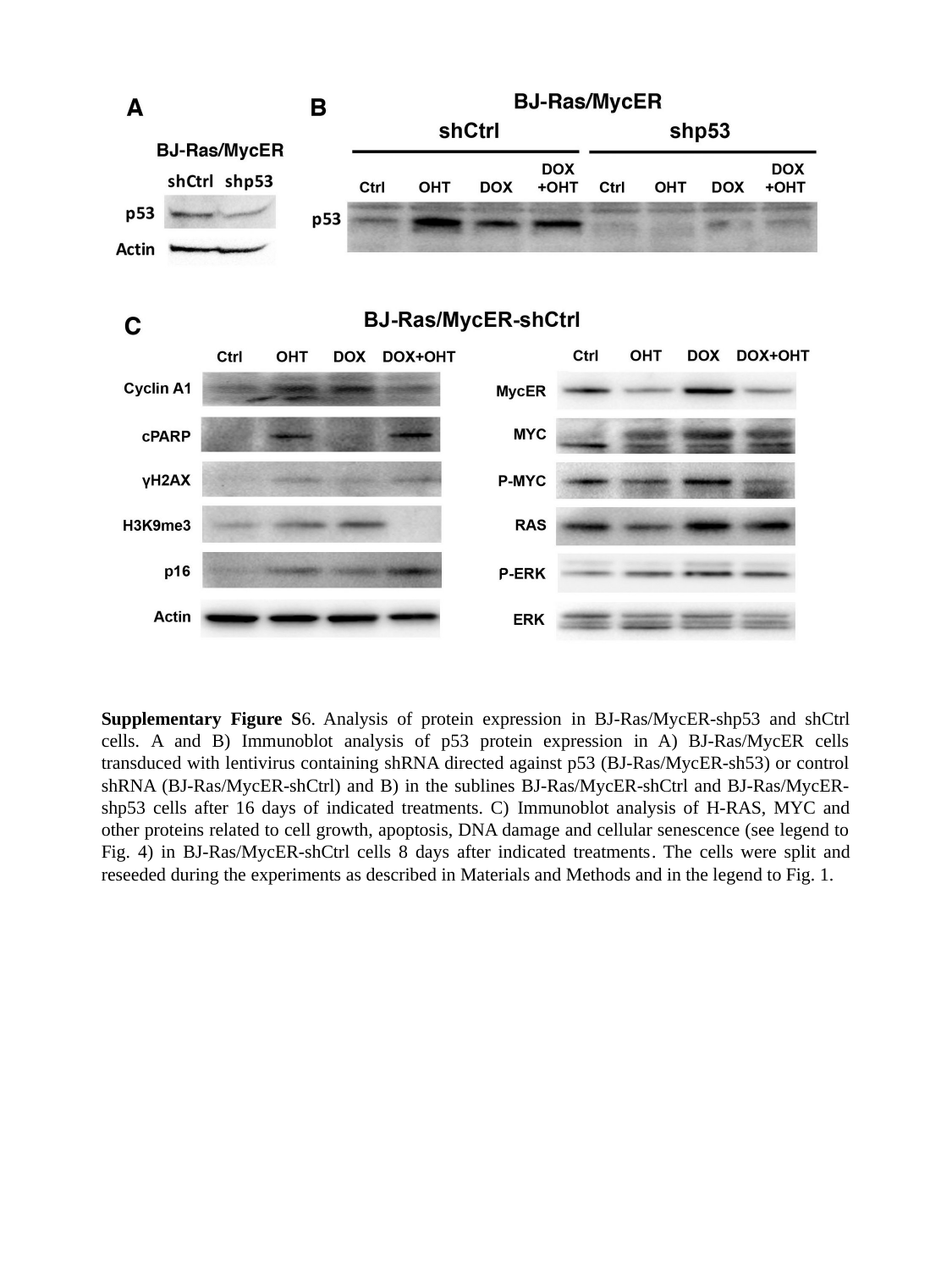

Supplementary Figure S6. Analysis of protein expression in BJ-Ras/MycER-shp53 and shCtrl cells. A and B) Immunoblot analysis of p53 protein expression in A) BJ-Ras/MycER cells transduced with lentivirus containing shRNA directed against p53 (BJ-Ras/MycER-sh53) or control shRNA (BJ-Ras/MycER-shCtrl) and B) in the sublines BJ-Ras/MycER-shCtrl and BJ-Ras/MycER-shp53 cells after 16 days of indicated treatments. C) Immunoblot analysis of H-RAS, MYC and other proteins related to cell growth, apoptosis, DNA damage and cellular senescence (see legend to Fig. 4) in BJ-Ras/MycER-shCtrl cells 8 days after indicated treatments. The cells were split and reseeded during the experiments as described in Materials and Methods and in the legend to Fig. 1.

## Slide 7
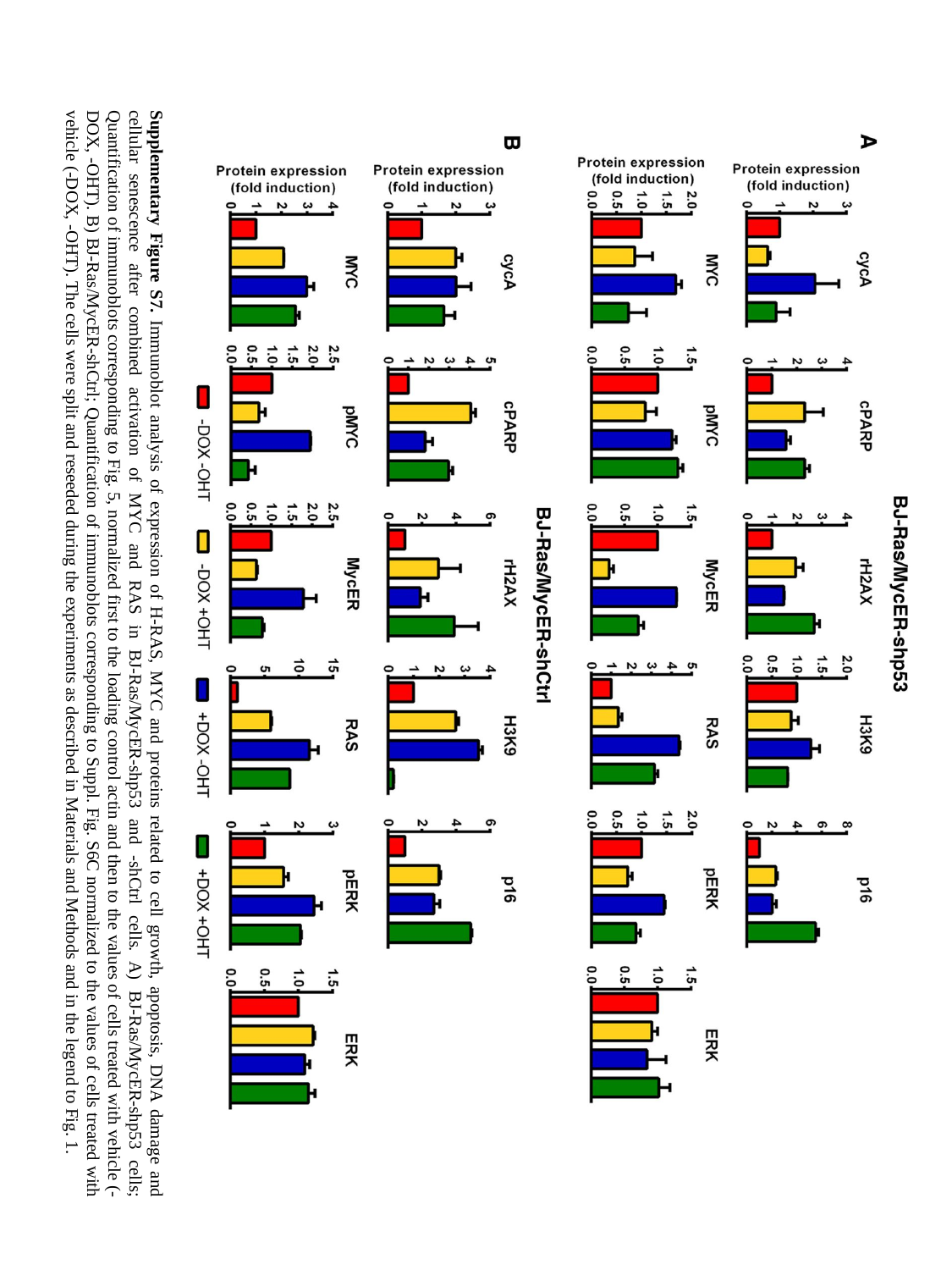

Supplementary Figure S7. Immunoblot analysis of expression of H-RAS, MYC and proteins related to cell growth, apoptosis, DNA damage and cellular senescence after combined activation of MYC and RAS in BJ-Ras/MycER-shp53 and -shCtrl cells. A) BJ-Ras/MycER-shp53 cells; Quantification of immunoblots corresponding to Fig. 5, normalized first to the loading control actin and then to the values of cells treated with vehicle (-DOX, -OHT). B) BJ-Ras/MycER-shCtrl; Quantification of immunoblots corresponding to Suppl. Fig. S6C normalized to the values of cells treated with vehicle (-DOX, -OHT). The cells were split and reseeded during the experiments as described in Materials and Methods and in the legend to Fig. 1.
